# Direct Viral RNA Detection of SARS-CoV-2 and DENV in Inactivated Samples by Real-Time RT-qPCR: Implications for Diagnosis in Resource Limited Settings with Flavivirus Co-Circulation

**DOI:** 10.3390/pathogens10121558

**Published:** 2021-11-29

**Authors:** Zhan Qiu Mao, Mizuki Fukuta, Jean Claude Balingit, Thi Thanh Ngan Nguyen, Co Thach Nguyen, Shingo Inoue, Thi Thu Thuy Nguyen, Le Khanh Hang Nguyen, Noboru Minakawa, Kouichi Morita, Thi Quynh Mai Le, Futoshi Hasebe, Meng Ling Moi

**Affiliations:** 1 Institute of Tropical Medicine, Graduate School of Biomedical Sciences, Nagasaki University, Nagasaki 852-8523, Japan; jxcdcmzq@126.com (Z.Q.M.); bb55420106@ms.nagasaki-u.ac.jp (M.F.); jcpbalingit@gmail.com (J.C.B.); pampanga@nagasaki-u.ac.jp (S.I.); minakawa@nagasaki-u.ac.jp (N.M.); moritak@nagasaki-u.ac.jp (K.M.); rainbow@nagasaki-u.ac.jp (F.H.); 2 Department of Virology, National Institute of Hygiene and Epidemiology, Hanoi 10000, Vietnam; thanhngan0605@gmail.com (T.T.N.N.); thachnc2000@gmail.com (C.T.N.); ticun_2002@yahoo.com (T.T.T.N.); nlkh@nihe.org.vn (L.K.H.N.); lom9@hotmail.com (T.Q.M.L.); 3 School of International Health, Graduate School of Medicine, the University of Tokyo, Tokyo 113-0033, Japan

**Keywords:** SARS-CoV-2, DENV, virus co-circulation, direct RT-qPCR, virus inactivation, biosafety

## Abstract

The RT-qPCR method remains the gold standard and first-line diagnostic method for the detection of SARS-CoV-2 and flaviviruses, especially in the early stage of viral infection. Rapid and accurate viral detection is a starting point in the containment of the COVID-19 pandemic and flavivirus outbreaks. However, the shortage of diagnostic reagents and supplies, especially in resource-limited countries that experience co-circulation of SARS-CoV-2 and flaviviruses, are limitations that may result in lesser availability of RT-qPCR-based diagnostic tests. In this study, the utility of RNA-free extraction methods was assessed for the direct detection of SARS-CoV-2 and DENV-2 in heat-inactivated or chemical-inactivated samples. The findings demonstrate that direct real-time RT-qPCR is a feasible option in comparison to conventional real-time RT-qPCR based on viral genome extraction-based methods. The utility of heat-inactivation and direct real-time RT-qPCR for SARS-CoV-2, DENV-2 viral RNA detection was demonstrated by using clinical samples of SARS-CoV-2 and DENV-2 and spiked cell culture samples of SARS-CoV-2 and DENV-2. This study provides a simple alternative workflow for flavivirus and SARS-CoV-2 detection that includes heat inactivation and viral RNA extraction-free protocols, with aims to reduce the risk of exposure during processing of SARS-CoV-2 biological specimens and to overcome the supply-chain bottleneck, particularly in resource limited settings with flavivirus co-circulation.

## 1. Introduction

The emergence of severe acute respiratory syndrome coronavirus 2 (SARS-CoV-2), a novel virus that causes severe respiratory symptoms, has recently caused a major global healthcare concern. COVID-19 has varied clinical presentations, with most of the DENV-infected individuals remaining asymptomatic or diagnosed as mild cases [1,2]. In severe and critical COVID-19 cases, mortalities are largely driven by severe respiratory failure related to interstitial pneumonia in both lungs and acute respiratory distress syndrome [3]. 

Many tropical and sub-tropical regions of the world where dengue outbreaks are seasonal, and where the recent dengue virus (DENV) epidemic occurred, are also facing the COVID-19 pandemic with many difficulties in terms of diagnosis [4]. Initial clinical features may be similar in both dengue and COVID-19, along with shared laboratory parameters such as thrombocytopenia and leucopenia [5]. Diarrhea and sore throat are also some common clinical features described in patients with either acute dengue or COVID-19 [6,7,8]. The similarity in clinical manifestations is of significant public health concern, especially in countries where dengue is endemic, as it remains a challenge to clinically differentiate dengue from COVID-19 at initial presentation. As dengue and COVID-19 require different clinical management, this may also pose a serious public health threat that may lead to adverse consequences, particularly in dengue-endemic countries. Hence, it is vital to strengthen the laboratory-based differential diagnosis of COVID-19 and flavivirus infections, particularly dengue fever, for proper management of critical patients. 

Early diagnosis of suspected cases is an important task in managing infected individuals and in controlling disease spread. Routine diagnosis of DENV infections is usually performed through quantitative reverse transcription polymerase chain reaction (RT-qPCR) by using serum samples. However, saliva and urine samples obtained during the acute phase could also be reliable alternative samples for DENV detection in the absence of serum samples as previously reported [9,10,11]. At present, the primary diagnostic method for COVID-19 is also RT-qPCR, which tests patient samples including nasopharyngeal swabs, sputum, and other lower respiratory tract secretions [12]. RT-qPCR is regarded as the gold standard for detecting RNA viruses, including SARS-CoV-2 and DENV, especially in the early stage of viral infection. By targeting a unique RNA sequence of the RNA virus, the genetic material of the pathogen can be directly detected by RT-qPCR.

SARS-CoV-2 RT-qPCR testing is vital in preventing disease spread between persons and communities that include asymptomatic cases, whose viral shedding can inadvertently spread infection to the elderly and people with certain medical conditions [13]. However, shortage of diagnostic reagents and supplies, such as PPEs [14] and RNA purification kits, remain major concerns in resource-limited settings that could lead to undertesting and underreporting of COVID-19 cases. To add to this complexity, majority of resource-limited countries that are currently burdened by the COVID-19 pandemic, also experienced concurrent dengue outbreaks further saturating their capacity for RT-qPCR testing and surveillance. A necessary step to solve this dilemma of limited nucleic acid diagnostic testing infrastructure is to develop easy-to-perform RT-qPCR protocols with quick turnaround time and limited equipment requirements. The increase in nationwide testing capacity between central and peripheral laboratories would considerably depend on safe and effective transportation and processing of samples allowing rapid and accurate laboratory diagnosis. 

Using clinical samples collected during the acute phase of SARS-CoV-2 and DENV-2 infections in Vietnam and Japan and, spiked cell culture samples of SARS-CoV-2, andDENV-2, this study evaluated alternative workflows for SARS-CoV-2 and DENV-2 RNA detection with and without either virus inactivation or nucleic acid extraction. Overall, this study demonstrated that the utility of inactivation methods and RNA extraction-free protocols potentially decreases user manipulation and assay time, as well as reduces the risk of sample contamination in areas with co-circulation of these viruses.

## 2. Materials and Methods

### 2.1. Ethics Statement

This study was approved by the Institutional Review Board of Institute of Tropical Medicine, Nagasaki University (EAN: 08061924-9, 170707205-3 and 200409233-3) and the National Institute of Hygiene and Epidemiology, Vietnam (IRB-VN01057-19/2019).

### 2.2. Cell Lines 

Baby hamster kidney cells (BHK-21, Japan Health Science Research Resource Bank) and African green monkey kidney Vero 9013 cells were maintained in Eagle’s minimum essential medium (EMEM, Gibco, Gaithersburg, MD, USA) supplemented with heat-inactivated 10% fetal calf serum (FCS) without antibiotics. All cell lines were cultured at 37 °C in a 5% CO_2_ incubator. 

### 2.3. Viruses

Two virus isolates were used in this study: (1) SARS-CoV-2 TY-WK-521/2020 strain (GenBank accession no. LC522975.1), isolated from a patient in Japan; and (2) Dengue virus type-2 (DENV-2) TL-30 strain (GenBank accession no. AB219135), isolated from a patient returning from Indonesia. SARS-CoV-2 TY-WK-521/2020 were propagated on Vero 9013 cells at 37 °C in 5% CO_2_ for 5 days, while DENV-2 TL-30 was propagated on BHK-21 [15]. Viral cell culture supernatants were collected, clarified by centrifugation, and stored in aliquots at −80 °C. Viral titers (plaque-forming units (PFU) per mL) were determined by plaque assay, and the viral genome copies per mL was determined by quantitative RT-qPCR.

### 2.4. DENV-2 Serum Samples and COVID-19 Nasopharyngeal Swab Samples

DENV-2 serum samples (*N* = 18) were collected from patients between April 2019 and September 2019 during the acute phase (3–5 days after initial onset of symptoms) in Vietnam. COVID-19 nasopharyngeal swab samples (*N* = 18) were obtained from clinics and public health facilities in Nagasaki prefecture, Japan. Nasopharyngeal swab samples were collected in 1.5 mL EMEM and SARS-CoV-2 infection was confirmed by real-time RT-qPCR. All SARS-CoV-2 and DENV-2 clinical samples were previously scored as positive by standardized testing conducted in the respective health facilities where the samples were obtained. For COVID-19 nasopharyngeal swab samples, they were previously scored positive by clinical diagnosis by on-site confirmation of viral genome by RT-qPCR. DENV-2 serum samples were stored at −80 °C pending analysis, and all frozen serum samples were thawed once for analysis.

### 2.5. Inactivation of Viruses in Cell Cultures and Clinical Samples

Clinical samples were inactivated by either adding an equal amount of DNA/RNA Shield 2X concentrate (Zymo Research, CA, USA) (chemical inactivation) or by heating at 95 °C for 10 min (heat inactivation). Solutions were subsequently processed for RNA extraction, and the presence of viral RNA was determined by real-time RT-qPCR. 

DENV-2 infected cell culture supernatants in EMEM with infectious titers of 10^1^ PFU/mL to 10^6^ PFU/mL were inactivated either by adding equal amount of DNA/RNA Shield 2X concentrate (Zymo Research, CA, USA) (chemical inactivation) or by heating at 95 °C for 10 min (heat inactivation). EMEM solutions were subsequently processed for RNA extraction and purification, and the presence of viral RNA was determined by real-time RT-qPCR. 

For the differential detection of SARS-CoV-2 and DENV-2, virus-infected cell culture supernatants in EMEM were spiked into 10-fold serial dilutions of SARS-CoV-2 or DENV-2 stocks with infectious titers of 5 × 10^1^ PFU/mL to 5 × 10^5^ PFU/mL at a 1:1 ratio. EMEM solutions were inactivated either by chemical or heat inactivation and RNA extracts were analyzed by real-time RT-qPCR as mentioned earlier. 

### 2.6. Viral RNA Extraction and Purification

Viral RNA was extracted and purified from 100 µL of both heat-treated and chemical-treated viral cell culture supernatants, swab samples and clinical samples using the Quick-Viral RNA Kit (Zymo Research, CA, USA), following the manufacturer’s protocol. The eluted RNA samples were either used immediately or stored at −80 °C pending analysis.

### 2.7. Quantification of DENV-2 and SARS-CoV-2 Viral RNA by Real-Time RT-qPCR

A range of ten-fold serial dilution of in vitro transcribed RNA from 10^3^ to 10^7^ PFU/mL was used to generate the standard curve [16,17]. Gene-specific primers and probes targeting the envelope (E) or nucleocapsid (N) gene for SARS-CoV-2 [16], and E gene for DENV-2 [17] were used (Supplementary Table S1). The viral RNA levels were defined as log10 viral genome copies per reaction. 

**Figure 1:**
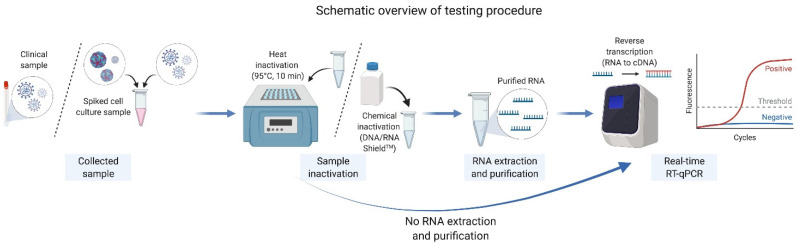
Schematic overview showing the workflow of the real-time RT-qPCR testing procedure used in this study.

Direct RT-qPCR (RNA extraction-free) and conventional RT-qPCR (with RNA extraction step) procedures were both performed in this study as represented on the schematic overview in Figure 1. Additionally, two types of RT-qPCR master mix were used for the quantitative real-time RT-qPCR assay: First, 20 µL real-time qPCR reaction mixture was prepared with TaqMan Fast Virus 1-Step Master Mix (Thermo Fisher), consisting of 5 µL of extracted RNA or serum sample, 5 µL of TaqMan™ Fast Virus 1-Step Master Mix (Applied Biosystems, CA, USA), 0.25 µL of 100 µM forward and reverse primer, 0.5 µL of 10 µM probe and 9 µL of nuclease free water. The experiment and the following real-time qPCR program on ABI instrument were set up as follows: 50 °C 5 min, 1 cycle; 95 °C 20 s, 1 cycle; 95 °C 3 s, 60 °C 30 s, 40 cycles.

Second, 20 µL real-time qPCR reaction mixture was prepared with Direct qPCR Master (JENA Bioscience, Jena, Germany), consisting of 2 µL of serum sample, 10 µL direct reaction mix, 0.16 µL of 100 µM forward and reverse primer, 0.40 µL of 10 µM probe, 0.8 µL enzyme mix, 6.48 µL of nuclease free water. Real-time qPCR program on ABI instrument was set up as follows: 50 °C 30 min, 1 cycle; 95 °C 5 min, 1 cycle; 95 °C 15 s, 60 °C 1 min, 45 cycles. Each mixture was added to the reaction to detect the amplification of target viral RNA (QuantStudio 7 Flex, Thermo Fisher Scientific, MA, USA). The real-time RT-qPCR results were analyzed with the QuantStudio™ Real-Time PCR Software ver. 1.1 and the amplification plots were reviewed for baseline and threshold values correction. 

### 2.8. Statistical Analysis 

Statistical analysis was performed using GraphPad Prism, version 8.4.3 (GraphPad, San Diego, CA, USA), with a 5% level of significance and two-tailed *p* values. Values were presented as mean. Logarithmic transformation of the data was carried out to obtain an approximately normal distribution of the viral genome copy values, and data were tested for normal distribution using the Shapiro-Wilk test. Log10 transformed viral genome copy values were analyzed either in two-group or multiple-group comparisons. Two-group comparisons were analyzed using Student’s *t*-test. Multiple group comparisons were analyzed by running both parametric (ANOVA) and non-parametric (Kruskal Wallis) statistical tests with Dunn’s and Tukey’s post-hoc tests. 

## 3. Results

### 3.1. Comparison of Chemical Inactivation and Heat Inactivation Real-Time RT-qPCR on COVID-19 Clinical Samples

To evaluate the real-time RT-qPCR method on heat-inactivated samples as a potential alternative diagnostic method, primer-probe sets targeting SARS-CoV-2 envelope (E) and nucleocapsid (N) genes were tested through chemical inactivation (ci-RT-qPCR) and heat inactivation (hi-RT-qPCR) methods using virus-infected cell cultures and nasopharyngeal swab samples from a COVID-19 patient after symptom onset. Both sample aliquots were serially diluted ten-fold, and sample dilutions were subsequently inactivated either by DNA/RNA Shield or by heating at 95 °C for 10 min. RNA extraction was then performed followed by real-time RT-qPCR. 

**Figure 2:**
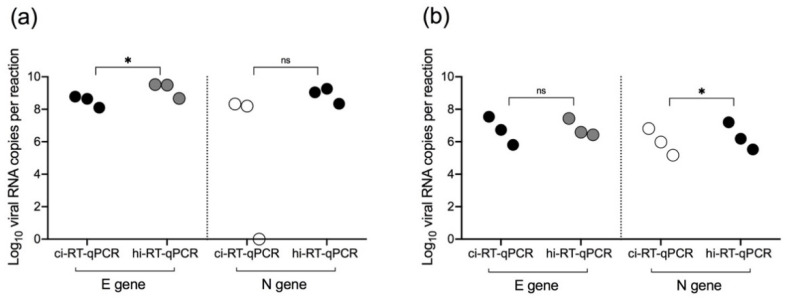
Comparison of chemical inactivation (ci-RT-qPCR) and heat inactivation (hi-RT-qPCR) real-time RT-qPCR on SARS-CoV-2 cell culture fluids and COVID-19 clinical samples using either E gene-specific or N gene-specific primer-probe sets. SARS-CoV-2-infected cell culture supernatants (**a**) and COVID-19 nasopharyngeal swab samples (**b**) were treated either by chemical (DNA/RNA Shield™) or heat (95 °C, 10 min) inactivation. SARS-CoV-2 E gene and N gene were amplified and detected by real-time RT-qPCR. Samples were analyzed in duplicates. Paired t test of log10 transformed viral RNA copies per reaction. Single asterisk (*) indicates a *p*-value of lesser than 0.05 (*p* < 0.04), and ns indicates a *p*-value of over 0.05 (*p* > 0.05).

Significant difference was observed between the heat-inactivated and chemical-inactivated cell cultures using E primer-probe set (Figure 2a, *p* = 0.013), but no significant difference using N primer-probe set (Figure 2a, *p* = 0.307). There was no significant difference between heat-inactivated and chemical-inactivated samples (Figure 2b, *p* = 0.674) using the E primer-probe set, while there was significant difference using the N primer-probe set (Figure 2b, *p* = 0.031). Given the relative sensitive performance of the E primer-probe set in COVID-19 nasopharyngeal swab samples (Figure 2b), the E primer-probe set was used for subsequent screening of acute COVID-19 clinical samples. 

**Figure 3:**
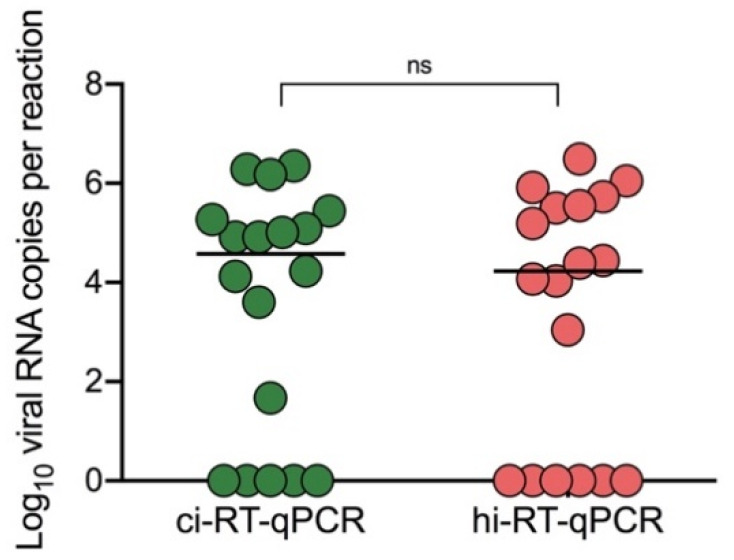
SARS-CoV-2 viral RNA copies in clinical samples between chemical- and heat-inactivated samples using real-time RT-qPCR. COVID-19 clinical samples (*N* = 18) were treated either by chemical (DNA/RNA Shield™) (ci-RT-qPCR) or heat (95 °C, 10 min) (hi-RT-qPCR) inactivation. SARS-CoV-2 E gene was amplified and detected by real-time RT-qPCR. Samples were analyzed in duplicates. Paired t-test of log10 transformed viral RNA copies per reaction between the detection methods was not significant, *p* > 0.05 (indicated as ns).

The optimized workflow was then evaluated using COVID-19 clinical samples (*N* = 18) which were previously scored as positive by clinical diagnosis by on-site confirmation of viral genome by RT-qPCR. Direct comparison between hi-RT-qPCR and ci-RT-qPCR was carried out. Heat-inactivated samples showed no significant difference on their viral RNA copy numbers with the chemical-inactivated samples (Figure 3, *p* = 0.363). Only one heat-inactivated sample was not detected, and the same sample showed comparatively low viral RNA copy number (46 copies per reaction) with high Ct value of 36.58 when the ci-RT-qPCR method was used. Heat-inactivated COVID-19 samples showed a ten-fold decrease in their viral RNA copy number as compared to the chemical-inactivated samples, with a 0.15 ± 0.67 (mean ± SD) difference. 

Additionally, the positive (PPA) and negative (NPA) percent agreements of the ci-RT-qPCR and hi-RT-qPCR were evaluated. For ci-RT-qPCR, 13 out of 18 clinical samples tested positive, while 5 showed undetermined results and were marked negative. For hi-RT-qPCR, 12 out of 13 samples showed positive results, while 5 out of 5 showed negative results. The results showed 92.3 % PPA and 100 % NPA (Table 1) demonstrating that hi-RT-qPCR can be an alternative method to ci-RT-qPCR. Given these findings, the results confirm that heat-inactivation of COVID-19 clinical samples could be a reliable procedure for SARS-CoV-2 RNA detection. 

### 3.2. Chemical and Heat Inactivation and Direct Real-Time RT-qPCR of Cell Culture-Propagated DENV-2

**Figure 4:**
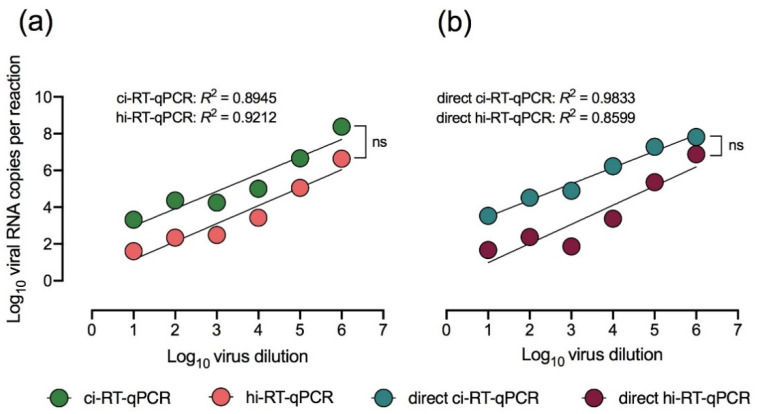
Linear regression of DENV-2 cell culture dilutions and viral RNA copy number using different real-time RT-qPCR methods. DENV-2-spiked samples were treated with chemical (DNA/RNA Shield™) (ci-RT-qPCR) or heat (95 °C, 10 min) (hi-RT-qPCR) inactivation and, DENV-2 E gene was detected either by (**a**) conventional real-time RT-qPCR or (**b**) direct (RNA extraction-free) real-time RT-qPCR (direct RT-qPCR). All serially-diluted samples were run in duplicates. Linear regression analysis of log10 transformed viral RNA copies per reaction with comparison of slopes and intercepts, *p* > 0.20. A *p*-value of over 0.05 (*p* > 0.05) is indicated as ns.

After determining the utility of hi-RT-qPCR for the detection of SARS-CoV-2 in acute COVID-19 clinical samples, the utility of this method on DENV-2 infected cell culture sample was further explored. Ten-fold serial dilutions of DENV-2 with infectious titers of 10^1^ PFU/mL–10^6^ PFU/mL were prepared for direct real-time RT-qPCR (RNA extraction-free) using chemical-inactivated (direct ci-RT-qPCR) and heat-inactivated (direct hi-RT-qPCR) cell cultures. The linear correlation coefficients (R^2^) of the methods tested consistently ranged from 0.80–0.98 showing good linear curve fitting (Figure 4). The overall slope was identical on all the real-time RT-qPCR methods tested. However, the viral RNA copy numbers of hi-RT-qPCR were consistently lower than that of the ci-RT-qPCR, as shown by differences in the elevation or intercept of the lines, suggesting that heat inactivation of DENV-2 could lead to lower levels of RT-qPCR sensitivity. 

Comparison of DENV-2 direct ci-RT-qPCR with the ci-RT-qPCR showed a ten-fold increase in the viral RNA copy number, with a difference of 0.39 ± 0.39 (mean ± SD) between the two methods. The results of direct ci-RT-qPCR and ci-RT-qPCR were nearly similar demonstrating that eliminating RNA extraction did not affect viral detection. 

### 3.3. Heat Inactivation and Direct Real-Time RT-qPCR of DENV-2 Clinical Samples

**Figure 5:**
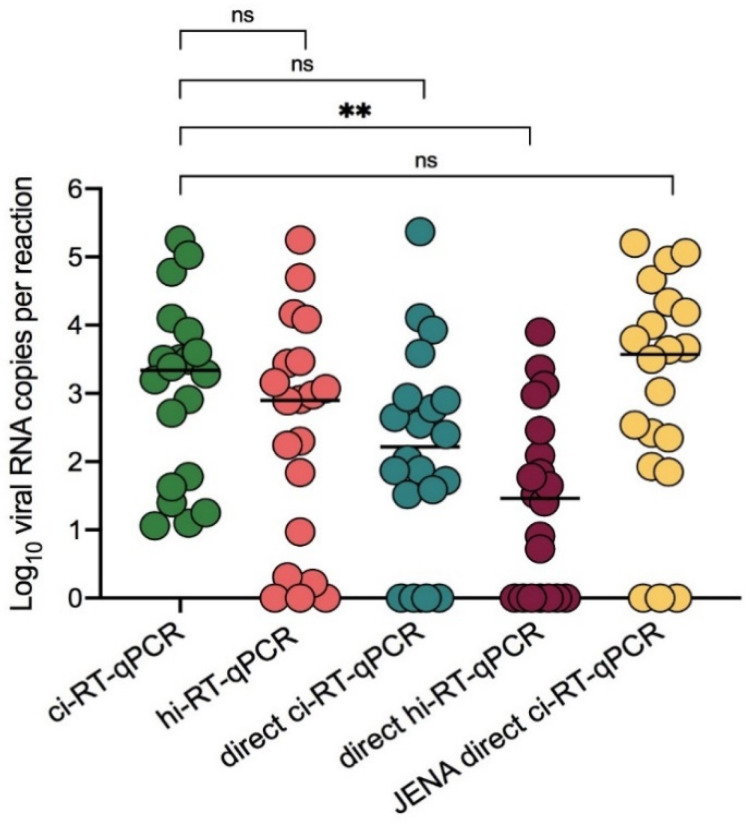
DENV-2 viral RNA copies in serum samples between chemical- and heat-inactivated samples using conventional and direct (RNA extraction-free) real-time RT-qPCR assays. DENV-2 serum samples (*N* = 20) were treated either by chemical (DNA/RNA Shield™) or heat (95 °C, 10 min) inactivation, and DENV-2 E gene was amplified and detected by conventional and direct real-time RT-qPCR (direct RT-qPCR) methods. Comparisons between DENV-2 RNA detection among chemical-inactivated (ci-RT-qPCR), heat-inactivated (hi-RT-qPCR), chemical-inactivated (direct ci-RT-qPCR), heat-inactivated (direct hi-RT-qPCR) and JENA RT-qPCR mix (JENA direct ci-RT-qPCR) was performed. Samples were analyzed in duplicates. Paired t-test of log10 transformed viral RNA copies per reaction, *p* > 0.05. One-way ANOVA Dunn’s multiple comparisons of log10 transformed viral RNA copies per reaction, ** *p* < 0.002. Double asterisks (**) indicates a *p*-value of lesser than 0.05 (*p* < 0.04), and ns indicates a *p*-value of over 0.05 (*p* > 0.05).

To further determine the utility of hi-RT-qPCR and direct RT-qPCR, DENV-2 clinical samples (*N* = 20) were tested. Heat-inactivated DENV-2 clinical samples showed no significant difference with chemical-inactivated samples (Figure 5, *p* = 0.180). Additionally, direct hi-RT-qPCR of DENV-2 clinical samples also showed no significant difference with direct ci-RT-qPCR (Figure 5, *p* = 0.220). In comparison to the ci-RT-qPCR, both direct ci-RT-qPCR and direct hi-RT-qPCR demonstrated lower viral RNA copy number by ten-fold, with a difference of −0.85 ± 1.37, and −1.66 ± 1.15 (mean ± SD), respectively. The PPA and NPA of the real-time RT-qPCR methods utilized in this study were also evaluated. hi-RT-qPCR and JENA direct ci-RT-qPCR demonstrated PPA values of 90% and 85%, respectively (Table 2).

Although no significant difference was observed between the ci-RT-qPCR and direct ci-RT-qPCR (Figure 5, *p* = 0.341), the viral RNA genome levels detected was lower in DENV-2 clinical samples as compared to that of DENV-2 spiked samples for direct ci-RT-qPCR. A significant loss of sensitivity was observed following direct hi-RT-qPCR. To determine the utility of a direct RT-qPCR master mix of direct ci-RT-qPCR in DENV-2 clinical samples, JENA Bioscience Direct qPCR Master was used as master mix for direct ci-RT-qPCR. Interestingly, the JENA direct ci-RT-qPCR method showed no significant difference to that of the ci-RT-qPCR (Figure 5, *p* > 0.999), with a minimal difference of 0.01 ± 0.86 (mean ± SD). These findings suggested that JENA Bioscience Direct qPCR Master mix could be used as an alternative RT-qPCR reagent for direct ci-RT-qPCR of clinical samples. 

### 3.4. Differential Detection of Chemical-Inactivated SARS-CoV-2 and DENV-2 in Spiked Samples by Real-Time RT-qPCR

**Figure 6:**
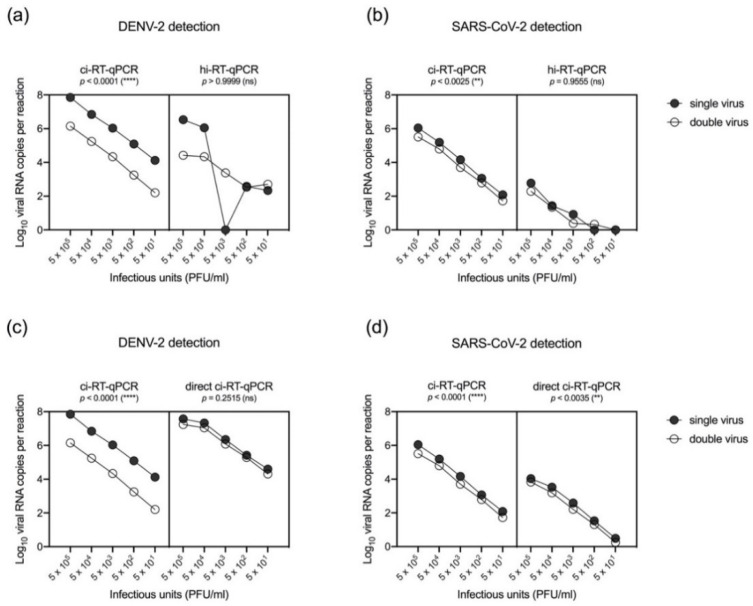
Differential detection of SARS-CoV-2 and DENV-2 in inactivated cell culture supernatants spiked with single virus (SARS-CoV-2 or DENV-2) and two viruses (double; SARS-CoV-2 and DENV-2) by using conventional or direct real-time RT-qPCR methods. Cell culture media spiked with standardized amounts of viruses were treated either by chemical (DNA/RNA Shield™) (ci-RT-qPCR) or heat (95 °C, 10 min) (hi-RT-qPCR) inactivation. DENV-2 E gene (**a**,**c**) and SARS-CoV-2 E gene (**b**,**d**) was determined either by conventional real-time RT-qPCR or direct real-time RT-qPCR (direct RT-qPCR). All serially-diluted samples were performed in duplicates. One-way ANOVA Dunn’s multiple comparisons of log10 transformed viral RNA copies per reaction.

To differentially detect SARS-CoV-2 and DENV-2, spiked samples in ten-fold serial dilutions of (1) DENV-2 or, (2) SARS-CoV-2, and (3) 1:1 ratio mix of DENV-2 and SARS-CoV-2 were inactivated by using either chemical or heat treatment. DENV-2 was detected in SARS-CoV-2-spiked samples either by ci-RT-qPCR or hi-RT-qPCR (Figure 6a) with lower RNA copies in spiked samples compared to single virus samples. Consequently, DENV-2 genome levels as detected by direct ci-RT-qPCR in spiked (DENV-2 and SARS-CoV-2) samples was also lower as compared to that of single virus samples (Figure 6c). However, no significant difference was observed between the spiked samples when direct ci-RT-qPCR was performed (*p* = 0.2515). Detection of SARS-CoV-2 in DENV-2-spiked samples was lower in hi-RT-qPCR and direct ci-RT-qPCR (Figure 6b or Figure 6d, respectively) compared to the ci-RT-qPCR results. The viral RNA copy numbers detected in either single-spiked DENV-2 or SARS-CoV-2 samples were higher than that of samples that were spiked with two viruses (DENV-2 and SARS-CoV-2) at the same time. DENV-2 viral genome was detected in spiked samples at lower detection limits of up to 5 × 10^1^ PFU/mL for both hi-RT-qPCR and direct ci-RT-qPCR, while SARS-CoV-2 was detected up to 5 × 10^1^ PFU/mL for direct ci-RT-qPCR and 5 × 10^3^ PFU/mL for hi-RT-qPCR. The results indicate that the detection sensitivity may be lower in spiked samples.

## 4. Discussion

Diagnostics play a fundamental role in the successful containment of outbreaks. Majority of developing countries that are currently burdened by the COVID-19 pandemic are also experiencing concurrent dengue outbreaks making it difficult to rapidly implement control measures and manage patient outcomes. The current diagnostic workflow for COVID-19 includes the collection of samples on-site, and subsequent transportation for molecular testing and sequencing. However, handling of SARS-CoV-2 remains a major concern in resource-limited countries due to limited access to diagnostic reagents and supplies such as PPEs [14], and inadequate diagnostic testing capacity at both national and community levels of healthcare. There is an urgent need for a safer and more efficient diagnostic workflow for SARS-CoV-2 and flaviviruses, like DENV, in countries where these viruses are co-circulating. To reduce the risk of exposure during transportation, handling, and testing, as well as aid in easier and faster sample processing, the utility of chemical and heat inactivation employed against SARS-CoV-2 and DENV-2 was assessed in this study. Additionally, the utility of RNA extraction-free protocols for the direct detection of SARS-CoV-2 and DENV-2 in inactivated samples using real-time RT-qPCR was also evaluated. 

In this study, the utility of both chemical inactivation and heat inactivation in the detection of SARS-CoV-2 RNA by real-time RT-qPCR was tested. The SARS-CoV-2 E primer-probe set was useful for both chemical and heat inactivation using COVID-19 nasopharyngeal swab samples. In concordance with a previous study that also used nasopharyngeal swab samples [18], heat inactivation decreased the sensitivity for N gene real-time RT-qPCR. Unlike other chemical inactivation methods that use lysis buffers (e.g., AVL and Trizol), this study used DNA/RNA Shield™ as an inactivating viral transport medium, as it can both inactivate and preserve viral RNAs and has been cleared for use as a transport medium for COVID-19 testing [19]. Both inactivation methods detected SARS-CoV-2 in acute COVID-19 clinical samples with no significant reduction in detection levels. Using two different inactivation methods, in DENV-2 samples, both methods detected viral RNA up to 5 × 10^1^ PFU/mL, with heat inactivation having minimal impact on real-time RT-qPCR detection levels. The results suggested that these inactivation methods have the potential to reduce the risk of exposure while collecting, transporting, and processing samples with little impact on detection rates for DENV and SARS-CoV-2. As such, the inactivation methods could present a more practical method, especially in resource-limited settings, due to its simplicity in sample handling and as it does not require purchase of additional diagnostic reagents, particularly in regions with co-circulation of flaviviruses and SARS-CoV-2. 

Apart from inactivation methods, the utility of direct real-time RT-qPCR on chemical- and heat-inactivated samples for the detection of SARS-CoV-2 and DENV-2 RNA was also determined. Conventional RT-qPCR uses extracted RNA and as such, the viral RNA extraction step is typically needed for RT-qPCR [20,21,22]. However, the viral RNA extraction step is expensive, laborious, and time-consuming, and represents a major bottleneck in diagnostic testing particularly during the peak of the COVID-19 epidemic [16]. While viral RNA extraction is useful for downstream application such as sequencing, in clinical settings during outbreaks, the viral RNA extraction step may limit the number of tests that can be done due to limited resources. Commonly used protocols developed for the detection of the novel SARS-CoV-2 require the use of RNA extraction kits [16,23,24,25]. The employment of RNA extraction in the current diagnostic workflow of COVID-19 not only constitutes significant time delay in sample processing, but also the shortage of reagent supply reducing large-scale testing in most countries. It is therefore crucial to explore other rapid, efficient, and scalable diagnostics assays for screening and surveillance. In this study, real-time RT-qPCR-based testing of SARS-CoV-2, and other representative flaviviruses like DENV-2 can be performed without the use of RNA extraction kits, demonstrating the possible utility of a workflow without the RNA extraction step. Chemical or heat-inactivated clinical samples could immediately be subjected to real-time RT-qPCR without intermediate steps. In this study, to further improve the sensitivity of the direct real-time RT-qPCR assay, the utility of a direct RT-qPCR mix (JENA Direct qPCR Master mix) was used with chemical-inactivated DENV-2 clinical samples. The qPCR Master mix reduces the potential inhibition of clinical samples. There were no significant differences between the levels of RNA detected by using JENA cid-RT-qPCR and the conventional direct ci-RT-qPCR, indicating that this direct qPCR method was compatible for clinical samples at comparable levels with that of conventional real-time RT-qPCR. Notably, loss in sensitivity following direct hi-RT-qPCR was observed as reported in a previous study [26]. Heat inactivation likely causes RNA fragmentation; hence it is important to use primer-probe sets that target shorter amplicons in direct hi-RT-qPCR to detect these RNA fragments.

One of the major points of this study is the detection of both DENV-2 and SARS-CoV-2 in the same RT-qPCR reaction. In the differential detection of SARS-CoV-2 and DENV-2 in spiked samples by RT-qPCR using chemical and heat-inactivated viral cell culture supernatants, DENV-2 was detected in DENV-2 and SARS-CoV-2-spiked cell culture samples. However, SARS-CoV-2 was detected at lower levels as compared to DENV-2 in the two viruses spiked cell culture samples. This indicates lowered sensitivity of E gene-based real-time RT-qPCR in heat-inactivated spiked viral cell culture supernatants, which was observed when using serially diluted viral cell culture supernatants (Figure 2a). The results indicated that heat inactivation and direct real-time RT-qPCR of spiked samples reduced the levels of detectable SARS-CoV-2 RNA in samples with two viruses (DENV-2 and SARS-CoV-2). As there were no clinical samples with SARS-CoV-2 and DENV-2 in this study, the differential detection of SARS-CoV-2 and DENV-2 in clinical samples could not be performed. However, saliva samples may provide an alternative method as a non-invasive method for sample collection [27,28]. Saliva-based technique has the efficacy of DENV RNA detection, in the acute phase of infection, with a high sensitivity rate of 87.5% by using saliva samples [29]. The viral RNA was detected up to 14 days after onset of symptoms by RT-qPCR in saliva samples [30]. In line with the results of the SARS-CoV-2 and DENV spiked samples, further studies using saliva samples of patients with co-infection, will provide insights on the utility of the alternative diagnostic testing for both of DENV and SARS-CoV-2 infections by using saliva samples [31,32]. While the Ct values were higher for each DENV-2 and SARS-CoV-2 in spiked samples with these two viruses, the results indicate that the assay may still be useful in the detection of DENV and SARS-CoV-2 infection. As the phenomenon may not be limited to this study, the results imply that further studies to optimize SARS-CoV-2 and DENV RT-qPCR should be done to determine the sensitivity in the detection of viral RNA in patients with SARS-CoV-2 and DENV co-infection by using saliva samples or other respiratory specimens. As such, saliva or respiratory samples may be useful in settings where invasive blood collection procedures are not available, or in resource-limited settings where nasopharyngeal samples were the only samples collected.

This study describes a streamlined method for the detection of SARS-CoV-2 and DENV-2 RNA from viral cell culture supernatants and clinical samples, however some limitations should be considered. This study was mainly performed using viral cell culture supernatants and a few numbers of clinical samples for evaluation, which may have limited better interpretation of the proposed workflows. This study was also not able to test other DENV serotypes and other mosquito-borne viruses such as the Japanese encephalitis virus and Chikungunya virus, due to sample unavailability. Testing the feasibility of detecting SARS-CoV-2 with these other circulating mosquito-borne viruses is also a significant parameter for determination in future studies. Additionally, the spiked samples used in this study were derived from viral cell culture stocks, hence this may be different from true co-infected samples, which may in turn create bias when evaluating the clinical performance of the methods proposed in this study. 

## 5. Conclusions

Overall, this study provides straightforward, cost-effective, and simpler alternative workflows for the detection of SARS-CoV-2 with cocirculating DENV-2 in resource-limited countries, by using viral cell culture supernatants and clinical samples. Although the data is limited and there is still a need to refine the procedures, the findings suggest that the heat inactivation and direct real-time RT-qPCR methods described in this study have the potential in reducing the risk of exposure during sample processing and may be useful in overcoming the supply chain bottleneck, particularly in resource-limited countries where SARS-CoV-2 and flavivirus infections are co-circulating.

## Figures and Tables

**Table 1:** Quantitative outcome of parallel testing of paired non-heat inactivation and heat inactivation on COVID-19 clinical samples.

		hi-RT-qPCR		Total
		Positive	Negative	
ci-RT-qPCR	Positive	12	1	13
	Negative	0	5	5
Total		12	6	18
* PPA = 92.3%; ** NPA = 100%				

* positive percent agreement (PPA) and ** negative percent agreement (NPA).

**Table 2:** Quantitative outcome of parallel testing of paired five methods on DENV-2 clinical samples.

		hi-RT-qPCR			cid-RT-qPCR			hid-RT-qPCR			JENA cid-RT-qPCR		
		+	−	Total	+	−	Total	+	−	Total	+	−	Total
ci-RT-qPCR	+	18	2	20	16	4	20	15	5	20	17	3	20
	−	0	0	0	0	0	0	0	0	0	0	0	0
Total		18	2	20	16	4	20	15	5	20	17	3	20
		* PPA = 90%			PPA = 80%			PPA = 75%			PPA = 85%		

* positive percent agreement (PPA).

## Data Availability

The data sets generated and/or analyzed during the current study are available from the corresponding authors on reasonable request.

## Supplementary Materials

The following are available online at https://www.mdpi.com/article/10.3390/pathogens10121558/s1, Table S1. Oligonucleotide primers and fluorogenic probes used in the SARS-CoV-2 and DENV-2 real-time RT-qPCR assays.
