# Supplementary material for: Direct Viral RNA Detection of SARS-CoV-2 and DENV in Inactivated Samples by Real-Time RT-qPCR: Implications for Diagnosis in Resource Limited Settings with Flavivirus Co-Circulation
Source: Pathogens. 2021 Nov 29;10(12):1558. doi: 10.3390/pathogens10121558 (PMC8705679; doi:10.3390/pathogens10121558)
Supplement: Supplementary file 1 [file pathogens-10-01558-s001.zip › pathogens-1468590-supplementary.pdf]

Table S1. Oligonucleotide primers and fluorogenic probes used in the SARS-CoV-2 and DENV-2 real-time RT-qPCR assays.

Virus	Primer and probes	Nucleotide sequence (5′ → 3′)	Target gene
SARS-CoV-2	Forward Primer	CACATTGGCACCCGCAATC	N (Sarbeco)
	Reverse Primer	GAGGAACGAGAAGAGGCTTG	
	Probe	FAM-ACTTCCTCAAGGAACAACATTGCCA-BHQ	
	Forward Primer	ACAGGTACGTTAATAGTTAATAGCGT	E (Sarbeco)
	Reverse Primer	ATATTGCAGCAGTACGCACACA	
	Probe	FAM-ACACTAGCCATCCTTACTGCGCTTCG-BHQ	
DENV-2	Forward Primer	ACACCACAGAGTTCCATCACAGA	E
	Reverse Primer	CATCTCATTGAAGTCNAGGCC	
	Probe	FAM- CGATGGARTGCTCTC-BHQ	
